# Management of gingival hyperpigmentation induced by increased ferritin level in a HbS‐β^+^ thalassemia patient using diode laser

**DOI:** 10.1002/ccr3.8171

**Published:** 2023-11-06

**Authors:** Raneem Darkazali, Omar Hamadah

**Affiliations:** ^1^ Department of Oral Medicine, Faculty of Dental Medicine Damascus University Damascus Syria; ^2^ Higher Institute of Laser Research and Applications Damascus University Damascus Syria

**Keywords:** depigmentation, diode laser, gingival pigmentation, HbS‐β^+^ thalassemia, serum ferritin

## Abstract

Diode lasers can be employed for the treatment of gingival hyperpigmentation in HbS‐β^+^ thalassemia patients due to the advantages that lasers provide including good hemostatic effect and less postoperative complications.

## Graphical abstract

We herein report a case of a sickle cell β^+^ thalassemia patient that had gingival hyperpigmentation which safely treated using diode laser, after following prophylactic procedures.

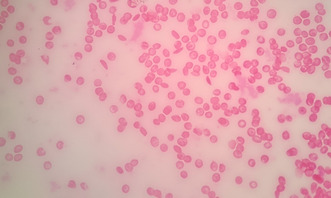

## 1 INTRODUCTION

Sickle cell β thalassemia or HbS‐β thalassemia is a rare form of sickle cell anemia, in which there is a coinheritance of sickle cell gene and β thalassemia gene.^1^, ^2^ The diagnosis of the disease can be made depending on family history, complete blood count and hemoglobin electrophoresis.^3^ According to HbA level, this genotype can be classified as: sickle cell β^0^ thalassemia with the absence of HbA, and sickle cell β^+^ thalassemia that is associated with HbA production.^4^ The severity of the disease is variable and depends primarily on the degree of the beta‐thalassemia mutation: decrease or absence of HbA.^5^ Sickle cell β^+^ thalassemia has a milder course, due to HbA synthesis. It may present with no symptoms, and can be misdiagnosed as iron deficiency anemia.^6^

All these factors can cause increased iron levels primarily in the form of ferritin including ineffective erythropoiesis, excessive gastrointestinal iron absorption due to hemolysis, and frequent red blood cells transfusion.^7^, ^8^ Many studies had shown that ferritin levels and pigmentations of skin and gingiva are correlated especially in thalassemia patients or those who undergo blood transfusion regularly.^9^, ^10^ In addition, it was found that iron deposition can enhance melanin production, which will lead to hyperpigmentation.^11^

Due to the rarity of sickle cell β^+^ thalassemia genotype, oral and maxillofacial manifestations are not well described; in addition, there is a lack in the literature regarding dental considerations when treating such patients.

Gingival hyperpigmentation has various causes; however, they can be classified as physiologic and pathologic factors. Many treatment modalities were used to manage gingival pigments, including traditional surgical techniques (bur abrasion or scalpel),^12^ electrosurgery,^13^ cryosurgery,^14^ and laser techniques.^15^, ^16^

Here we report a case of a female patient with HbS‐β^+^ thalassemia that presented with mild symptoms of the disease, but is being regularly undergone blood transfusion, she was referred to the department of oral medicine with a chief complaint of unaesthetic appearance of dark gingiva, the patient underwent laser surgery to remove the gingival pigmentations.

## 2 CASE PRESENTATION

**FIGURE 1:**
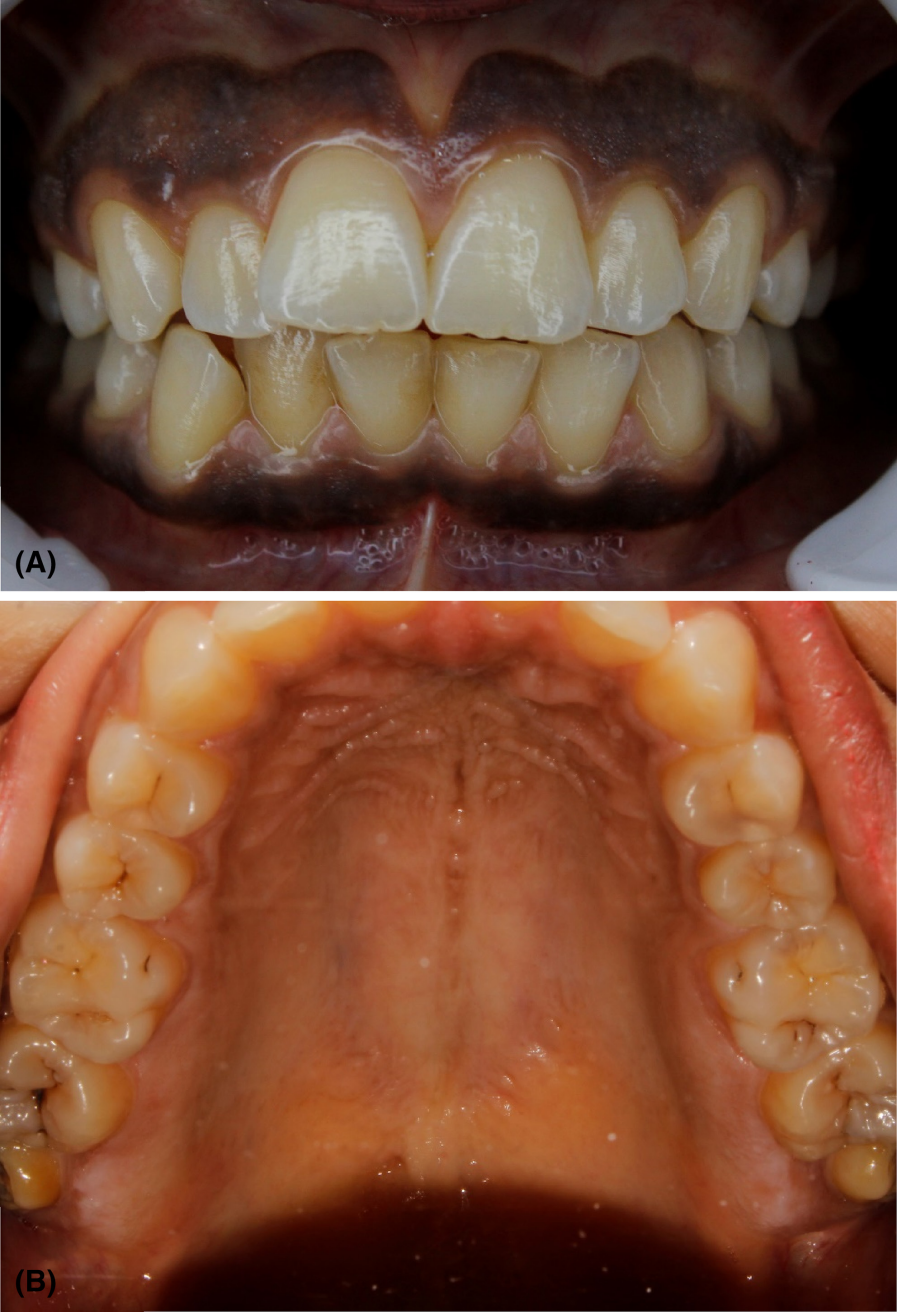
(A) Deep brown gingival pigmentation, (B) mild brown pigmentation in the hard palate.

**FIGURE 2:**
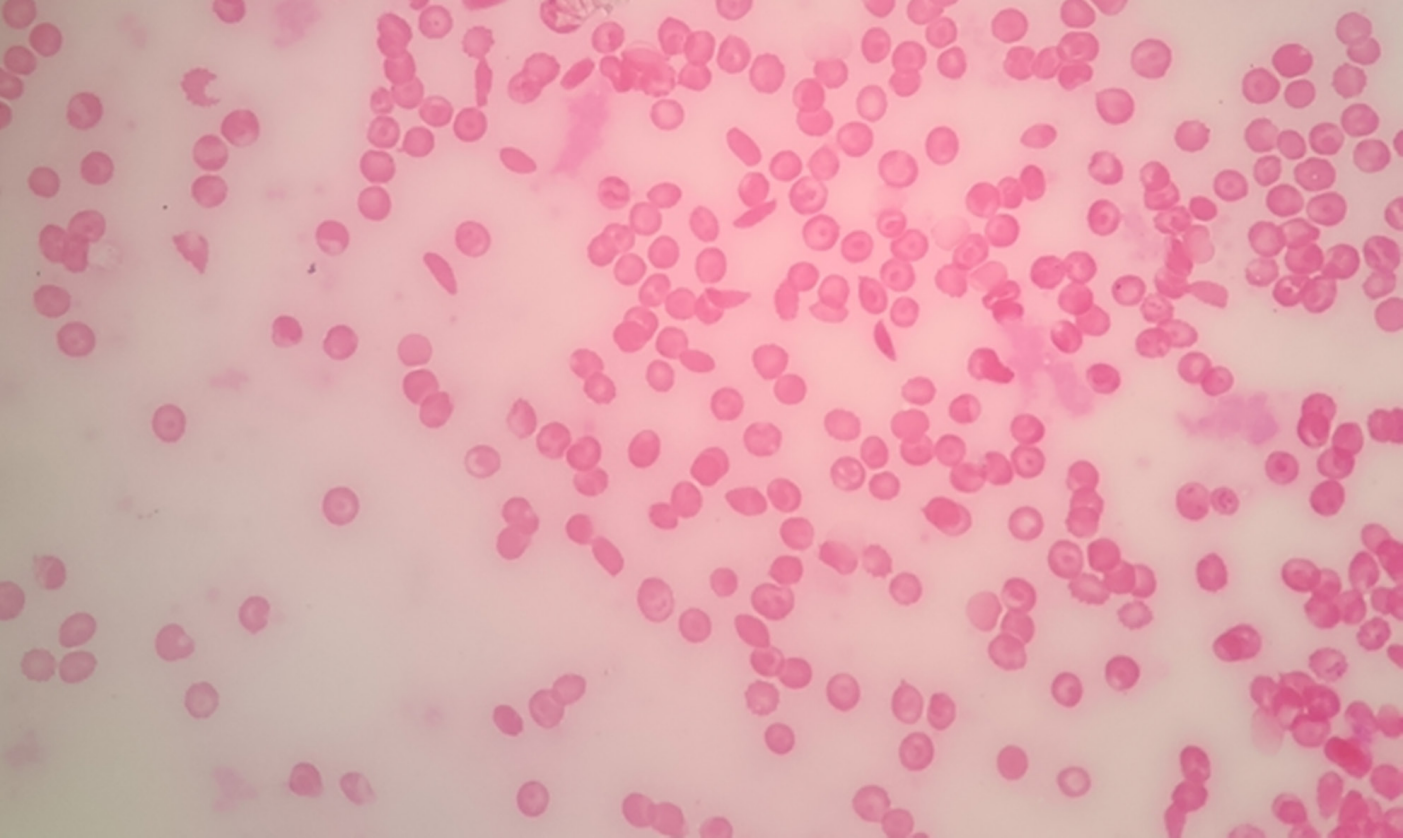
Blood smear showing sickling cells and target cells.

A 21‐year‐old female patient was referred to oral medicine department in the faculty of dental medicine at Damascus university, with a chief complaint of unaesthetic appearance of hyperpigmented gingiva and mild pigmentation in the hard palate (Figure 1). She was previously diagnosed with sickle cell β^+^ thalassemia (hemoglobin electrophoresis results are shown in Table 1), and blood smear is shown in Figure 2, with a history of splenectomy in the childhood. The patient was given blood transfusion every 4 months, to correct the hemoglobin levels, and was under deferiprone to treat iron overload. Ferritin level at first session was 4000 μg/L. The patient reported no vaso‐occlusive crises, only headache and mild joints pain. Extraoral examination revealed paleness of face skin. Intraoral examination showed good oral hygiene, no signs of periodontal diseases or active carious lesions. Only paleness of oral mucosa, black gingival pigmentation (score 3 according to Dummet et al.^17^), and light brown pigmentation in the hard palate were noticed. According to the patient's clinical, medical, and family history, and laboratory findings, she was diagnosed with gingival pigmentations induced by increased ferritin level.

**TABLE 1:** Demonstrates hemoglobin electrophoresis results after 4 months of the last blood transfusion.

Hemoglobin type	Fractions (%)
HbA	13.4
HbF	4.50
HbS	78.8
HbA2	3.3
HbC	0

Hematological consultation was performed before any intervention. It revealed that the health condition of the patient permits the surgery, since hemoglobin level is ≥8 g/dL. Antibiotics prophylaxis was recommended before the laser surgery. Local anesthesia without epinephrine was recommended. The patient's blood values before 3 days of the laser surgery are indicated in (Table 2).

**TABLE 2:** The patient's blood values after 1 day of blood transfusion and 3 days' prior laser surgery.

Blood test	Result
White blood cells (WBC)	12 × 10^+3^/μL
Red blood cells (RBC)	3.24 × 10^+6^/μL
Hemoglobin (HBG)	9.9 gm/dL
Mean corpuscular volume (MCV)	91 fl
RDW‐CV	17.5%
Platelets	687 × 10^+3^/μL
Hematocrit (HCT)	29.5%
Mean corpuscular hemoglobin (MCH)	30.6 PG
Mean corpuscular hemoglobin concentration (MCHC)	33.6 gm/dL

**FIGURE 3:**
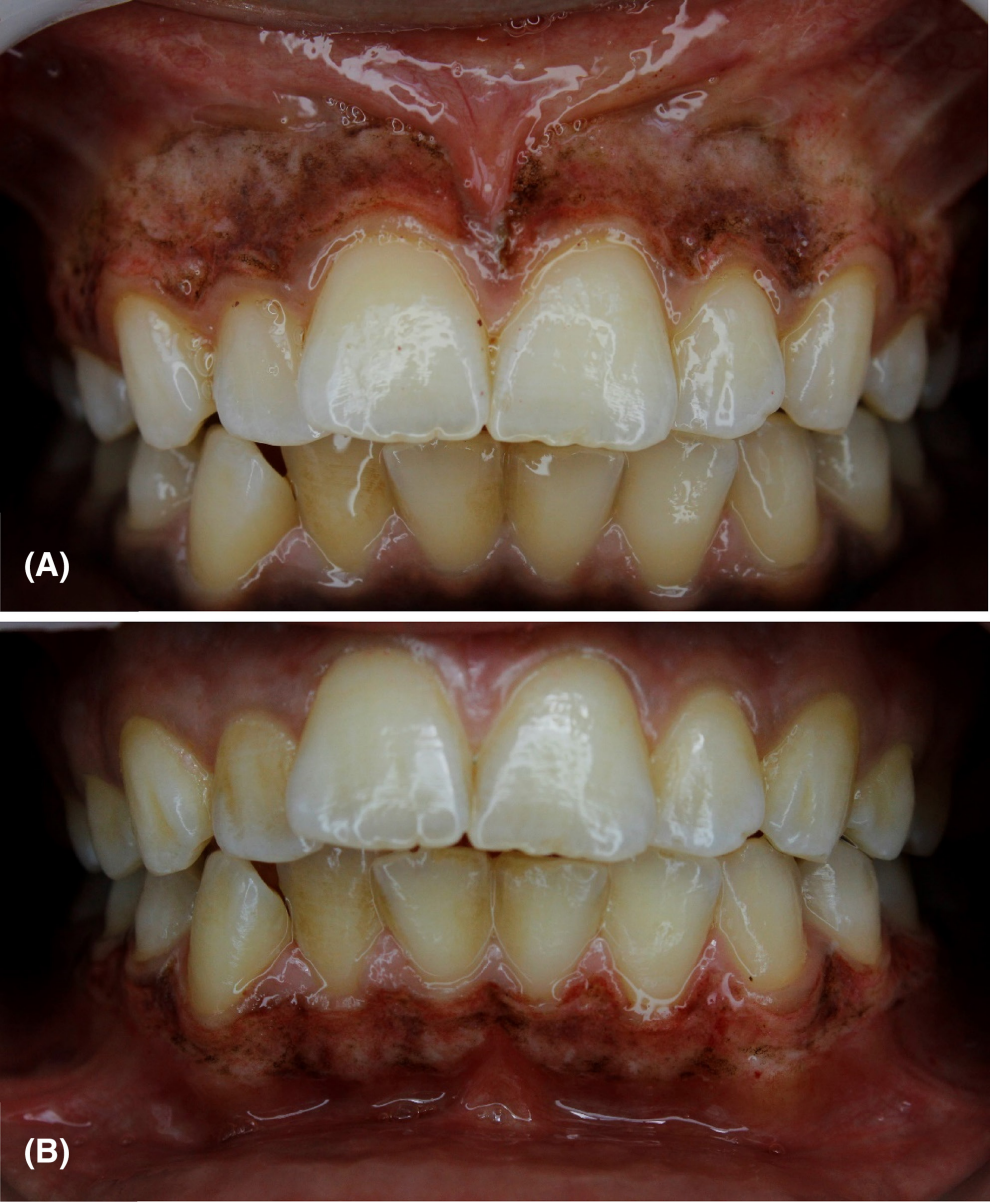
Direct postoperative views of gingival depigmentation in maxilla (A) and mandible (B).

Amoxicillin (1 g) was prescribed before 30 min of treatment. After sufficient local anesthesia using mepivacaine, the diode laser was used to remove the gingival pigmentations using the following parameters (pulsed mode, average power: 0.8 W, peak power: 1.6 W, duty cycle: 50%). The gingival depigmentation needed two sessions (one session for each jaw). Gingival epithelial ablation was accomplished using short light paint brush strokes and a gauze soaked in saline to remove the epithelium debris. Depigmentation was done from right second premolar to left second premolar in each jaw. Each treatment session took 12–15 min. Figure 3 shows direct postoperative views of gingival depigmentation in both maxilla and mandible. Laser provided hemostasis and clear work field. Amoxicillin 500 mg every 8 h and paracetamol 500 mg in case of need were prescribed. The patient was instructed to avoid trauma, and spicy and acidic foods during the first 5 days after treatment.

**FIGURE 4:**
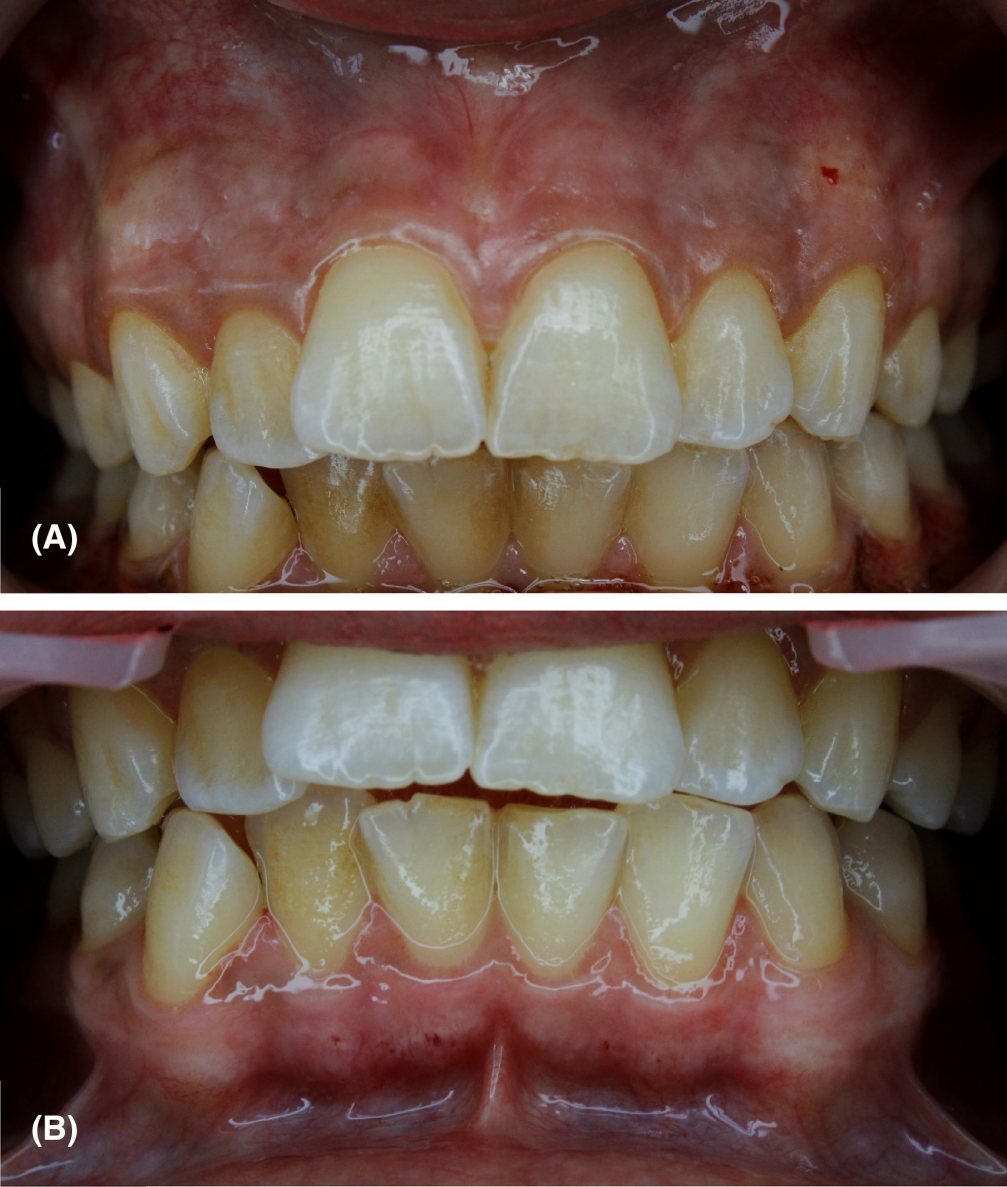
Ten days postoperative views (A) maxilla, (B) mandible.

**FIGURE 5:**
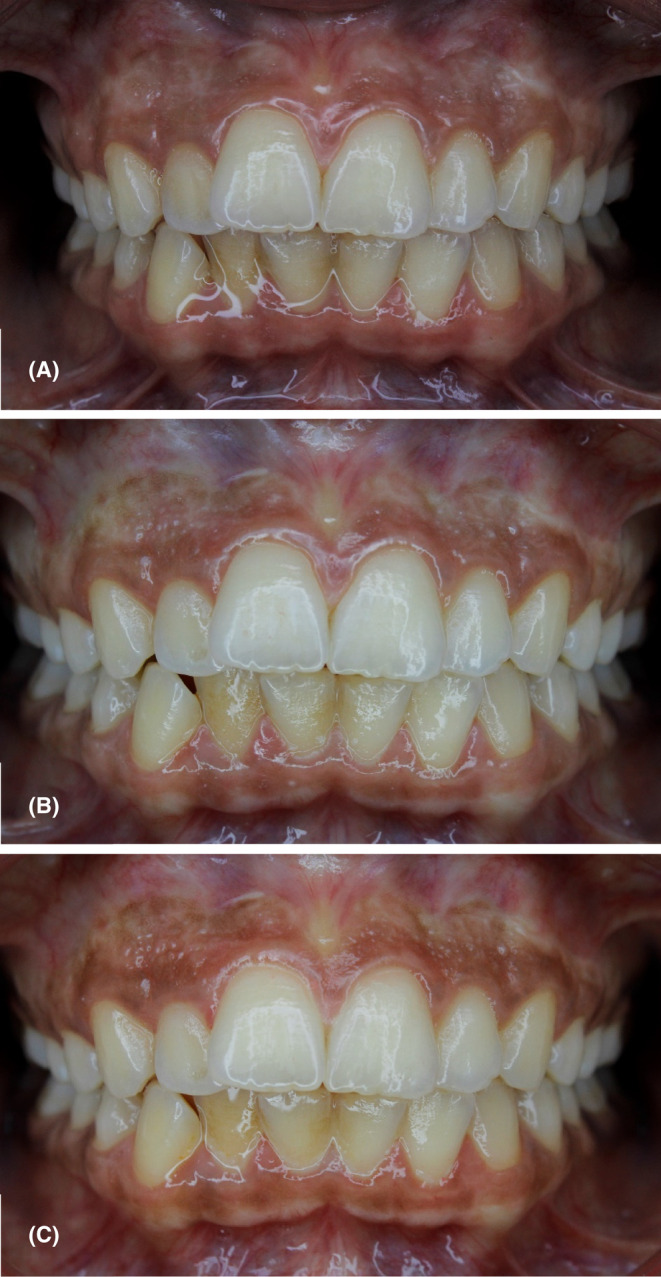
Follow‐up pictures after the completion of treatment sessions, (A) 1‐month postoperative view showing complete healing of gingiva with no signs of gingival recession or pigmentation recurrence, (B) 3 months postoperatively showing mild recurrence of the pigmentations in some areas with significant paleness of gingival tissues, (C) 6 months' postoperative view showing mild recurrence of pigmentation in nearly entire gingival tissues.

No bleeding, swelling, or wound infection were reported. She had a moderate pain in the first 2 days, which became milder in the third day, and disappeared 5 days postoperatively. The patient was followed after 10 days of each treatment session (Figure 4), 1, 3, and 6 months after the completion of treatment sessions (Figure 5).

## 3 DISCUSSION

This is a case of gingival pigmentation induced by increased ferritin level that was treated successfully and safely using the diode laser in a patient with sickle cell β^+^ thalassemia, which is considered a rare variant of SCD.

According to HbA levels, HbS‐β^+^ thalassemia also can be sorted in three groups: type I: 1%–7% HbA; type II: 7%–14% HbA; and type III 14%–25% HbA.^18^, ^19^ Variable amounts of HbA alleviate HbS, thus inhibit cellular damage caused by polymerization, consequently higher amounts of HbA associated with milder phenotype.^19^, ^20^ Also, it was found that the rate and extent of polymerization can be modified by the erythrocyte HbF concentration.^21^, ^22^ All these reasons can explain the mild course of the disease in this patient.

Gajjar and his colleagues studied the association of ferritin levels and gingival and skin pigmentation degree in beta thalassemia patients. They had concluded that gingival hyperpigmentation and skin color changes had been seen among older multi‐transfused patients, also had noticed a significant relation between ferritin levels and gingival pigmentations and skin color changes.^10^

Although good amounts of data are available regarding the diagnosis and management of sickle cell disease, however, there is a lack in the literature concerning management of this rare phenotype. Antibiotic prophylaxis was recommended for this patient. It was shown that patients who had underwent splenectomy are at infectious risk.^23^ Also it was found that sickle cell disease increases the susceptibility to infection.^24^ It is important to obtain deep anesthesia to avoid situations of stress which may induce subsequent occlusive vascular pain.^25^ The most preferred safe analgesic treatment for sickle cell anemia patients is a combination of paracetamol and codeine,^26^ in this patient, paracetamol was sufficient to alleviate the pain after laser surgery.

Gingival hyperpigmentation can have a negative effect on patient's psychosocial status, and can be a considerable concern for many patients.^27^ In this case, the 21‐year old female insisted on the treatment of the gingival pigments which were causing a cosmetic concern.

Laser is an effective, reliable, and comfortable treatment modality. It can provide many advantages including excellent hemostatic effect, clear work field, and short chair time. Moreover, it was shown that laser techniques associated with less postoperative complications such as pain, bleeding, discomfort, and infection when compared to conventional surgical techniques.^16^, ^28^, ^29^, ^30^, ^31^ Also, it is required to apply periodontal pack after gingival depigmentation using conventional techniques, whereas, when using lasers, there is no need for it, because a white fibrin slough will appear in the first days following the procedure, which will serve as a biological wound dressing that seals the ends of sensory nerves.^32^

## 4 CONCLUSION

The Diode Lasers can be employed for the management of gingival hyperpigmentation in HbS‐β^+^ thalassemia patients. It can provide many advantages including excellent hemostatic effect, and reduced postoperative complications. Hematologist consultation is essential to assess the health condition of the patient. This case was treated after following prophylactic procedures based on the patient's disease characteristics.

## AUTHOR CONTRIBUTIONS

**Raneem Darkazali:** Data curation; investigation; methodology; writing – original draft. **Omar Hamadah:** Investigation; methodology; supervision; writing – review and editing.

## FUNDING INFORMATION

The authors have no sources of funding.

## CONFLICT OF INTEREST

The authors declare that they have no competing interests.

## ETHICS APPROVAL STATEMENT

Ethical approval was obtained from ethical review committee of Damascus university.

## CONSENT

A written consent was obtained from the patient to publish clinical details and photographs.

## Data Availability

The datasets generated during this study are available from the corresponding author on a reasonable request.
